# China’s environmental policy intensity for 1978–2019

**DOI:** 10.1038/s41597-022-01183-y

**Published:** 2022-03-11

**Authors:** Guoxing Zhang, Yang Gao, Jiexun Li, Bin Su, Zhanglei Chen, Weichun Lin

**Affiliations:** 1 School of Management, Lanzhou University, Lanzhou, 730000 China; 2 Institute of Green Finance, Lanzhou University, Lanzhou, 730000 China; 3 Department of Decision Sciences, Western Washington University, Bellingham, WA 98225 USA; 4 Energy Studies Institute, National University of Singapore, Singapore, 119620 Singapore; 5 Department of Industrial Systems Engineering and Management, National University of Singapore, Singapore, Singapore

**Keywords:** Climate-change policy, Politics, Government

## Abstract

Improving the measurement of environmental policy intensity would affect not only the selection of variables in environmental policy research but also the research conclusions when evaluating policy effects. Because direct evaluation is lacking, the existing research usually applies data such as pollutant emission data, or the number of policies to construct proxy variables. However, these proxy variables are affected by many assumptions and different selection criteria, and they are inevitably accompanied by endogeneity problems. In this study, China’s environmental policy is comprehensively collected for the first time, and a machine learning algorithm is applied to evaluate the policy intensity. We provide all the policies issued by the Chinese government from 1978 to 2019 and the quantified intensity for each policy. We also distinguish all policies into three types according to their attributes. This dataset can help researchers to further understand China’s environmental policy system. In addition, it provides a valuable dataset for related research on evaluating environmental policy and recommending actions for further improvement.

## Abstract (summary)

**Table:** 

Measurement(s)	China’s environmental policy intensity
Technology Type(s)	machine learning algorithm

## Background & Summary

Environmental issues have become a global challenge that threatens the health and livelihood of humans and all living creatures on earth. In particular, during decades of rapid industrialization, China has been suffering from an environmental crisis, including air pollution, water scarcity, etc. These issues, in return, have limited the sustainability of economic and social development in China^1^. Over recent decades, China’s fight against environmental problems has advanced through the implementation of a series of environmental policies. These policies have achieved significant successes as indicated by the reduction in energy intensity and pollutant emissions. However, which policies or which parts of the policies have led to success? It would be of tremendous value to study the relationships between environmental policies and their outcomes: this would require a quantitative analysis of policies.

Quantitative analyses of environmental policies directly affect the assessment of environmental policy outcomes, providing the ability to continuously monitor policy effectiveness and improve the environmental policy system. China has proposed the goals of “carbon peaking” and “carbon neutral”, which will inevitably set higher requirements for future environmental policies and lead to increasingly complex policies being promulgated^2,3^. With the emergence of big data and large samples, it is essential that suitable ideas and methods be found to improve quantitative research on policies.

Policy instruments are described as “building blocks” to transfer the rather abstract principles and rules into concrete and substantive action^4^. In the research on policy change^5,6^, policy outcomes^7,8^, policy outputs^9^, policy mix^10^, many researchers have focused on the importance of policy instruments. For example, Knill *et al*.^8^ proposed measuring policy intensity by six indicators: objectives, scope, integration, budget, implementation, and monitoring. These indicators form a content-based coding procedure that allows the systematic assessment of policy intensity over time and space as well as across policy fields^10^.

Policy intensity has also emerged as a powerful tool for quantitative policy research. Policy intensity is an index that weights policy instruments according to measures such as whether the instrument has measurable targets, designated budgets, clear objectives and timelines; its integration with larger policy initiatives; and the enactment of policy monitoring. In the literature, there are several related concepts, such as “importance”, “significance”, or “stringency^11–13^”. For example, the Organization for Economic Co-operation and Development (OECD) has developed an index of environmental policy stringency^14–16^. Based on these definitions, policy intensity in this paper is the degree of stringency in the delivery and output of a policy document. The higher the policy intensity of a document is, the more stringency there is confronting the policy stakeholders.

Quantitative analysis of policy often involves a significant amount of manual reviewing, measuring and annotating comprehensive indices from policy text^17,18^. For environmental policy, empirical studies rely on a variety of regulatory impact data^19–23^. In recent years, a growing number of studies have suggested the use of policy output data to assess the influence in a more direct fashion^24^. Terms such as policy strength, policy objectives, policy measures have gradually attracted more attention^25–29^. However, the processes of collecting and coding policy instruments are not sufficiently systematic and are somewhat fragmented. The manual process is often tedious and time consuming. It is challenging to ensure the efficiency and accuracy of quantification methods when facing a large collection of policy documents. More importantly, policy assessment is heavily dependent on policy resources^8^. To the best of our knowledge, there has been no study that systematically organizes and discloses a comprehensive collection of environmental policy data in China.

To fill those gaps, we build a comprehensive collection of environmental policies in China over 40 years and develop a novel and systematic method to assess the policy intensity quantitatively. This indicator provides an excellent way to understand and appreciate China’s environmental policy system. The quantitative results would help researchers to systematically observe the change and development of China’s environmental policies over time. In addition, our dataset provides a detailed inventory of environmental policies, allowing researchers to reduce the labour and time costs of collecting environmental policies in China. Our dataset will facilitate the advancement of environmental policy-related research, and researchers can use it further to develop a systematic assessment of China’s environmental policies.

## Methods

**Fig. 1:**
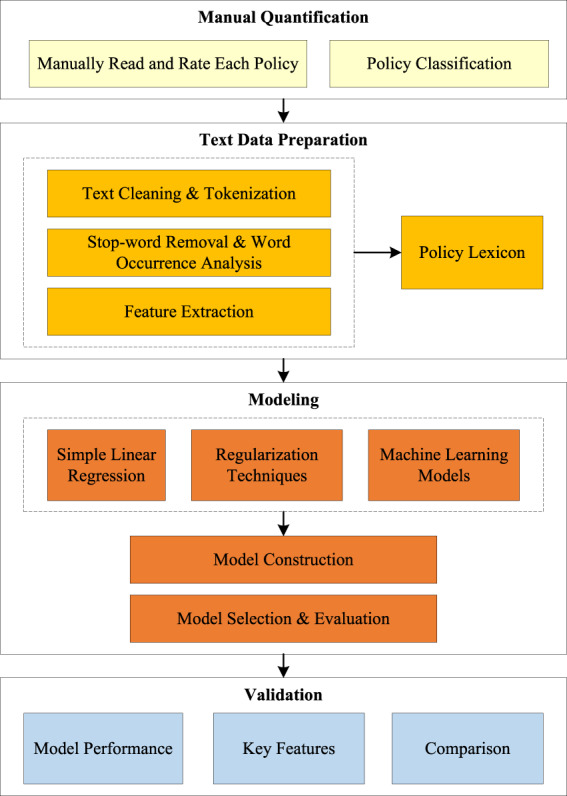
Research framework. (1) *Manual Quantification*: we first manually read and score each policy. Then, we categorize policies into different types according to their attributes. (2) *Text Data Preparation*: We analyze the text content by a series of text-mining operators, e.g., cleaning, tokenization, stop-word removal, word frequency analysis, and feature extraction from the text. The specific lexicon of environmental policy is screened and constructed. (3) *Modeling*: We deploy a series of prediction models to quantify the intensity of environmental policies. (4) *Validation*: To validate the credibility of the results, we evaluate and compare models based on their performance and key features.

An overview of our methods is shown in Fig. 1. The research framework consists of four modules: Manual quantification, Text data preparation, Modeling, and Validation.

### Data collection

We first collect environmental policies from the Global Legal and Regulatory Network (http://policy.mofcom.gov.cn/), China Legal Resources Database (http://www.lawyee.org), Wanfang Database (http://c.g.wanfangdata.com.cn), China National Knowledge Infrastructure (CNKI, https://www.cnki.net/), PKULaw.com database (https://pkulaw.com/), the official websites of the China and its ministries, etc. Keywords such as “energy savings,” “emissions reduction,” “energy conservation,” “reducing pollutant emissions,” “pollutant,” “low carbon,” and “energy” were used to search and collect environmental policies jointly or independently promulgated by the National People’s Congress and the State Council from 1978 to 2019.

Then, the policy text was carefully read considering the aspects of policy background, release date, issuing institution, policy type, policy objectives, and policy measures. After a long period of collation, classification, discussion, and screening, a dataset of China’s environmental policies was finally established. More than 40 agencies jointly or independently promulgated 1912 environmental regulation policies in the dataset. Some of the departments that promulgated policies are shown in Table 1.

**Table 1:** List of departments that have promulgated environmental policies.

**Departments that promulgate environmental policies (list only a portion of all departments)**	
The National People’s Congress	The Ministry of Supervision
The State Council	The Ministry of Agriculture
The Ministry of Ecological Environment	The Forestry Bureau
The National Development and Reform Commission	The State Administration of Taxation
The Ministry of Finance	The China Banking Regulatory Commission
The Ministry of Transport	The Electricity Regulatory Commission
The Ministry of Industry and Information	The State Administration for Industry and Commerce
The Ministry of Housing and Urban-Rural Development	The State Council Administration of Organ Affairs
The Ministry of Science and Technology	The Ministry of Education

### Manual quantification

The manual quantification of environmental policy intensity mainly involved combinations with pre-set dimensions, such as policy objectives and policy measures. Through an interpretation of the policy text considering the enforceability and content details of different policy measures and objectives, each policy is rated on a scale of 1 to 5 in terms of its intensity^25,26^. Policy measures include personnel measures, administrative measures, fiscal and tax measures, financial measures, guiding measures, and other economic measures. Policy objectives include preventing and controlling pollution, improving the effectiveness of energy conservation and emission reduction, establishing awareness of energy conservation and emission reduction, promoting industrial upgrading, improving energy use efficiency, optimizing the energy consumption structure, and promoting the technological transformation of energy conservation and emission reduction.

We trained a group of personnel to manually read and rate each policy for the intensity of measures and objectives. Each policy was rated by multiple raters independently and validated for interrater reliability. The ratings not only reflect the degree to which the policy emphasizes certain measures or objectives but also to a certain extent solve the problem of weight selection in the process of constructing indicators. The formula for calculating the intensity of environmental policy is as follows:

1 $$\begin{array}{c}ERI={M}_{tik}{O}_{tin}\end{array}$$

where *t* represents the year, and *i* represents a policy. *M*_*tik*_ is the sum of the intensity of *k* policy measures in a certain year, and *O*_*tin*_ is the sum of the intensity of *n* policy objectives in a certain year.

These environmental regulation intensity (ERI) scores rated by our personnel can serve as a valuable and reliable resource for future research related to China’s environmental policy. Furthermore, these ERI scores, along with the policy text, can be used to train a machine learning model that can estimate the intensity of future environmental policies.

### Environmental policy types

The policy instrument is the “carrier” of policy, a channel through which policy science researchers can study the main content of policy, the policy formulation process and the policy tools, and it serves as an objective, accessible, traceable written record of the policy system and policy process^30,31^. Most research on environmental policy focuses on policy tools, policy types, and game behaviour among the central bodies of governments at all levels in implementation^32,33^. For example, the World Bank divides environmental policy tools into four types: environmental regulations, market application, market creation, and public participation^34^. Based on the driving mechanisms, environmental policy can be divided into command-control policy and market-based policy^35–41^.

In this paper, we followed related research and divided environmental policy into three types: *command-control environmental policy* (CCEP), *market-based environmental policy* (MBEP) and *public participation environmental policy* (PPEP). CCEP and MBEP have been extensively investigated in the literature. The main characteristic of CCEP is that it is mandatory, and thus it relies on administrative instruments such as certain types of governance and standards. By contrast, MBEP mainly uses market-based instruments for environmental governance, such as fiscal policies related to environmental governance, emissions fees, emissions trading, product promotion catalogues, etc.

PPEP refers to the public and private sectors that have the right to participate in environmental protection. The Environmental Protection Act implemented in 2015 stipulated the principle of “public participation” in the “General Regulations” section: “All organizations and individuals have the obligation to protect the environment and have the right to report and accuse the organizations and individuals who pollute or destroy the environment”^42^. Therefore, we consider an environmental policy in the category of *public-participation* if it contains terms such as “*public participation*”, “*citizens*”, “*opinions*”, “*seek advice*”, “*supervision*”, “*hearing*”, “*argument*”, and “*subject declaration*”.

### Pre-processing of the text data

The encoded information in the text is enriched and supplemented in traditional research with structured data. In recent years, there have been many studies based on text, such as financial news, social media, speeches by political and corporate documents, and so on^43–46^. The most widely used method in text analysis is the bag-of-words model based on the document-word matrix. Researchers have discussed the practical application effects of various word lists and specific words in the field^47^. However, as Loughran *et al*.^44^ pointed out, “many studies rely on classification dictionaries derived from other disciplines, and such applications may produce false results”. The selection or construction of a lexicon suitable for a specific field presents a problem that needs to be fully considered before research can commence.

To avoid this problem, a specific lexicon applicable to environmental policies was constructed during the processing of textual data. The text preprocessing process in this paper includes several steps, such as text cleaning, tokenization, and word frequency statistics. Since the term-document matrix contains all the words that appear in the policy documents and many words have little importance to policy intensity, it is necessary to screen the words before forming a specific lexicon:

- Words that appear in more than 99% and no more than 1% of the documents are screened and deleted;
- The correlation coefficients between word frequency and policy intensity (manual quantification intensity) are calculated;
- Based on the results of the coefficients, words with high correlation (>0.1) with the intensity are selected;
- The selected word is divided into two aspects, policy objectives and policy measures, and the words representing objectives and measures were not repeated;
- Words that are not substantive, such as the names of people, places, departments, etc., are removed.

### Machine learning methods

In the literature, the use of text language to calculate indicators is gradually increasing^48–50^. The combination of this method and machine learning tools also enables the testing and verification of corresponding prediction scores based on specific structures. For example, Harrison *et al*.^51^ introduced a new language-based method to measure the five personality traits of CEOs. We first use the text analysis method to construct a specific lexicon suitable for environmental policy. Second, we construct a model to explore the relationship between the lexicon and policy intensity. Because of the nature of the task, traditional measurement methods and machine learning algorithms can both be applied. In this paper, the traditional linear regression model is selected, and eight groups of algorithm models are selected, including Ridge (Ridge), Lasso (Lasso), robust linear model (RLM), partial least squares (PLS), generalized linear model (GLM), support vector machine (SVM), eXtreme gradient boosting (XgbLinear), and random forest (RF).

Ridge and Lasso are shrinkage methods for prediction models and are particularly useful for datasets with a large number of explanatory variables^52^. RLM and GLM relax the conditions of least squares regression and are better able to handle outliers or external variances in the data. PLS projects prediction variables and observation variables to a new space to find a linear regression; this method is suitable for the linear regression task with high-dimensional data. SVM is also suitable for dealing with high-dimensional data and finding fitted curves. XgbLinear and RF are mainly tree-based methods. When out-of-sample prediction ability is essential, these are both prevalent and effective methods for flexibly estimating regression functions^53^. Several of the representative algorithms selected have also achieved good performance in other fields, and can thus well support this research.

## Data Records

Our dataset contains a total of 1,912 environmental policies from 1978 to 2019, along with their intensity scores quantified by experts and machine-learning models. In particular, the dataset includes 1,391 CCEP policies, 292 MBEP policies, and 229 PPEP policies. All data records have been uploaded to the public data repository Figshare^54^, specifically including the following:

- Dataset of environmental policy intensity measurement results for China (1978–2019) [“China’s environmental policies intensity, 1978–2019”];
- Dataset of the Chinese environmental policy lexicon [“Featured words in China’s environmental policies”];
- Dataset of the importance of key variables [“Key variables importance”].

### Environmental policy intensity

**Fig. 2:**
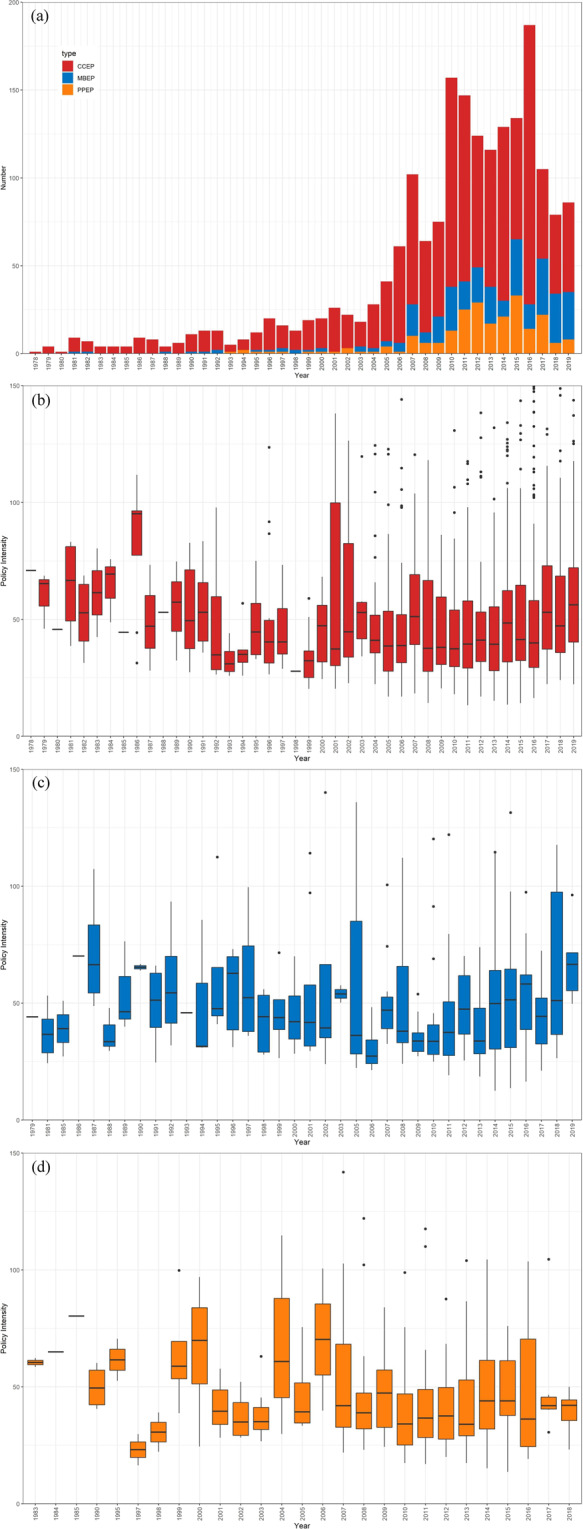
The distributions of policy types and policy intensity. The four subplots show: (**a**) the distribution of the three policy types, (**b**) box plots of CCEP policy intensity, (**c**) box plots of MBEP policy intensity, and (**d**) box plots of PPEP policy intensity.

This dataset provides the results over 40 years of China’s environmental policy intensity from Reform and Opening Up (1978) to 2019. Specifically, this dataset includes all environmental policies issued by the Chinese government every year and their corresponding policy intensity. The policy number (e.g., 2019–086) in the dataset reflects the number of environmental policies issued each year. Figure 2a–d show a comparison of the three policy types in terms of their policy count, mean and trend in the distribution of policy intensity by year, respectively. As shown in Fig. 2a, China has implemented an increasing number of environmental policies across all three types, especially during the last ten years, and the trend appears to be slowing down. CCEP (red) is still the predominant type of environmental policy in China.

As shown in Fig. 2b–d, the yearly variation in policy intensity is significant. Environmental policy intensity in earlier years shows greater volatility, and the degree of volatility decreases over time. In terms of the average trend in policy intensity, all policies have shown an upward trend in recent years. This trend reflects that China has been reinforcing its regulations to fight environmental issues via various types of policy. The difference between different types of policy is also becoming more visible, which reflects that China is adopting a differentiated strategy in the process of environmental governance.

### Environmental policy lexicon

We create a lexicon for quantifying the intensity of environmental policy in China that is included in the dataset. Specifically, we divide the words in this lexicon into two categories: policy objectives and policy measures. We also construct detailed subcategories under the objectives and measures categories. Table 2 provides the number of words in these categories.

**Table 2:** Number of words in the lexicon for measuring the intensity of environmental policy.

Category	Subcategory	Number of words
Policy Objectives	1. preventing and controlling pollution	23
	2. improving the effectiveness of energy conservation and emission reduction	30
	3. establishing awareness of energy conservation and emission reduction	19
	4. promoting industrial upgrading	39
	5. improving energy use efficiency	32
	6. optimizing the energy consumption structure	20
	7. promoting the technological transformation of energy conservation and emission reduction	28
	8. comprehensive goals	55
Policy Measures	1. personnel measures	22
	2. administrative measures	67
	3. fiscal and tax measures	16
	4. financial measures	4
	5. guiding measures	42
	6. other economic measures	21
	7. measure behavior	57

## Technical Validation

### Data retrieval and collection

In this research, we build a comprehensive collection of environmental policies issued by multiple levels of governments and ministries in China from 1978 to 2019 by retrieving policy documents from different official databases and websites. This process was carefully designed and executed to ensure comprehensive coverage and integrity in the data collection. Next, by carefully reading the policy text, we sort out those that are relevant to environmental regulation, which ensures the accuracy of data collection.

### Model performance

In this research, we empirically train and test learning-based models that allow automatic estimation of the intensity of environmental policy in China (from 1978 to 2016). To allow comparison, our experiments include several popular statistical learning algorithms, from simple linear regression models (LM) and regularization techniques (Ridge regression and LASSO) to machine learning models (e.g., SVM and random forest). For evaluation, we randomly split the entire data collection into a training set (75%) and a test set (25%). We apply each learning algorithm to the training set and estimate the training error using 10-fold cross validation. Then, we apply the trained prediction model to the test set and estimate the testing error. Table 3 summarizes the training and testing errors for the different prediction models. Among all models, RF achieved the best performance in terms of root mean squared error (RMSE) for both the training and test sets. Therefore, we choose RF to measure policy intensity.

**Table 3:** Performance of each model in the training and test sets.

Training Set									
	LM	Ridge	Lasso	RLM	PLS	GLM	SVM	XGBoost	RF
RMSE	72.98	51.78	46.03	71.17	47.05	45.82	47.23	42.03	38.71
**Testing Set**									
RMSE	99.84	61.28	54.06	97.28	41.58	52.91	40.93	35.63	34.23

### Identifying the key features

After showing that learning-based models can estimate the intensity of environmental policy, we assess the importance of features and identify the key indicators of policy intensity. The inquiry results based on the importance of variables can help to validate and interpret the prediction models. For evaluating variable importance, there are two types of methods: model-specific and model-agnostic^55^. Model-specific methods use certain elements of the model structure to evaluate the importance of variables. For example, in a linear regression model, the value of normalized coefficients and their corresponding p-values can be interpreted as reflecting the importance of variables, whereas in tree-based models, top decision nodes are considered more important variables for prediction. In contrast, without making any assumptions about the model, model-agnostic methods measure the impact on the model fitness when a variable (or a subset of variables) is removed from the model. Suppose $L=f\left(\widetilde{y},y\right)$ is the loss function of prediction model *f*(*x*). Then, the importance of variable *x*_*i*_ can be defined as:

2 $$\begin{array}{c}vi{p}_{Diff}\left({x}_{i}\right)={L}^{\ast i}-L\end{array}$$

or

3 $$\begin{array}{c}vi{p}_{Ratio}\left({x}_{i}\right)={L}^{\ast i}/L\end{array}$$

where *L* is the value of the loss function calculated by all variables *x*, and L^*i^ is that calculated by variables except *x*_*i*_.

In this research, for the different models we build for prediction, we use the model-agnostic method for evaluating the importance of variables. For each variable, we calculate the *vip* score by dropping it out of the model. “Data Record 3” summarizes the 20 most important variables in terms of contribution to the model.

In particular, for policy objectives, we can see that the variables relevant to energy and technology objectives play the most important roles, such as the optimization of the energy consumption structure (2 words belong to this category) and promoting the technological transformation of energy conservation and emission reduction (3 words belong to this category). For policy measures, administrative measures (3 words belong to this category), fiscal and tax measures (3 words belong to this category), and financial measures (2 words belong to this category) appear to play the most important roles.

Both policy objectives and policy measures have large impacts on policy intensity. The setting of objectives and corresponding measures are critical to environmental policymaking. Our results show that policies with more specific objectives and measures tend to have higher intensity. In particular, when the policy content involves details related to energy, technology, industry, fiscal and tax policy, finances, etc., the policy intensity is often higher.

To test the robustness of our findings, we used two additional metrics, i.e., mean square error (IncMSE) and node purity (IncNodePurity), to measure the importance of variables. Among the top 20 variables identified by IncMSE, nine belong to policy objectives: promoting industrial upgrades, optimizing the energy consumption structure, improving energy efficiency, establishing awareness of energy conservation and emission reduction, and comprehensive goals; and 11 variables belong to the policy measures, including measure behaviour, administrative measures, fiscal and tax measures, financial measures, and guiding measures. Among the top 20 variables identified by IncNodePurity, 14 belong to policy objectives, and six belong to policy measures. The results of IncMSE and IncNodePurity indicate that policy objectives have a more significant impact on policy intensity than measures. However, the specific objectives and measures, involving technological transformation, industrial upgrading, fiscal and tax measures, and others, continue to play an important role in policy intensity.

### Comparison with existing databases

To our knowledge, our dataset is the first large collection of environmental policies with their intensity scores estimated by both human experts and learning-based models. To evaluate the degree of difference between the two results (manual quantification and machine quantification), we objectively compare the results of the policy intensity from 1978–2016. From the perspective of sequence similarity measured by the shortest distance, the basic standard is that the closer the distance is, the higher the similarity. The classical measurement methods generally include two types: lockstep measures and elasticity measures^56^. Euclidean distance is a typical lockstep measure used to compare the point-to-point distance between time series^57^, and standardized Euclidean distance is an improvement of Euclidean distance, as the latter is affected by the inconsistency of the multidimensional data scale. Elasticity measures show an improvement compared with lockstep measures, as they can compare two time series “one to one” or “one to many”. Dynamic time warping (DTW) is a typical representative elastic measure that can calculate the similarity between time series and is especially suitable for time series of different lengths and rhythms. Compared with the traditional Euclidean distance, this method can describe the similarity of time series more accurately.

We select two indicators, standardized Euclidean distance and DTW, to compare the two time series of policy intensity scores manually rated by experts and those estimated by the learning-based models. The results show that the standard Euclidean distance is 0.05, and the DTW distance is 5.59, which indicates that the policy intensity estimated by the model is highly consistent with the results of expert scoring.

**Fig. 3:**
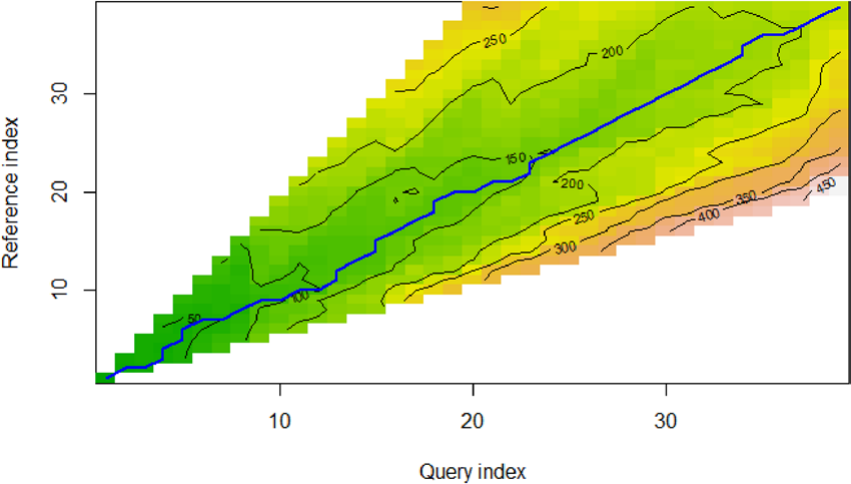
The neat path diagram with the minimum DTW distance between sequences.

By depicting the neat path that minimizes the DTW distance between sequences, the difference between the manually and machine-quantified intensity can be further judged (see Fig. 3). The blue line is the path with the lowest warping cost. When it coincides with the main diagonal or the gap is small, there can be considered to be high consistency between the two values. As seen from the results, the neat path of the model is relatively smooth, the cost of the neat path is small, and the neat path is parallel to the main diagonal. This shows that the intensity measured by the machine learning algorithms has a high degree of consistency with the results of manual quantification, reflecting the real changing trend in policy intensity.

### Limitations and future work

We quantify the policy intensity from policy text, and the analysis process and results have several implications for the application of text analysis and machine learning methods in economics and management. Simultaneously, the environmental policy lexicon constructed by the text analysis method can provide a reference for the quantitative study of policy intensity. We also provide the necessary dataset for research on environmental policy evaluation, which can help for researchers to systematically understand and evaluate China’s environmental policy system and will promote related researches on environmental policy. This dataset can greatly reduce the difficulty for researchers to conduct research on China’s environmental policy, and provide researchers with a booklet to find relevant policies conveniently and efficiently. Although our environmental policy dataset mainly includes the policies issued by the central government, it can be re-used and inspire further researches in several aspects:

- First, by quantifying the policy texts, we introduce the concept of “policy intensity” that, rather than simply indicating the existence of a policy, allows measuring environmental policies with a scale. Several studies have demonstrated the power of analyzing policy texts for analyzing environmental, social, and economic impacts^10,26^. Using our dataset and proposed methodology, policy analysts can study the evolution of policy intensity over time and gain insights into governments’ attitudes towards environmental issues at different time points. The comparison of the intensity of different policy types over years can also provide a basis for a more insightful analysis of environmental policies.
- Second, environmental policies are the documentation of the policy makers’ ideas and the basis of administration and execution. For their prior constraints and guidance nature, policies impose certain restrictions on stakeholders’ behaviors. While the policy intensity measured by the policy texts can indicate the strength of regulation, the quantification of the policy itself is only the starting point of evaluating environmental policy. At the end of the day, it is the execution of environmental policies that can directly determine the effects on the environment. Future research could look into regulatory actions in the policy execution process and measure the strength of these actions as well as their environmental impacts. By comparing the intensity of policy documents and their execution, we can gain more insights into the key factors to the success of environmental regulation.
- Third, from the perspective of local governments and industry, most environmental policies promulgated by provincial/local governments are largely aligned with those by the central government in China. Local policies often involve further refinement and enforcement of the policies from the above, without significant adjustments in essence. Hence, researchers can further investigate and design new comprehensive indicators of policy intensity, by combining our dataset with the strength of execution by local governments (e.g., the number of environmental enforcement officers, pollution emission monitoring level, or the degree of pollution control). Such an indicator could become an essential variable for evaluating the impact of environmental policies on company behaviors. In addition, researchers can apply our proposed analytical approach to collect and analyze more environmental policies from local governments or other countries and further expand the dataset.

## Supplementary information

Supplementary Information

## Data Availability

The relevant code data used for calculation and analysis in this paper can be obtained from the supplementary information.
