# Author Correction: China’s environmental policy intensity for 1978–2019

**DOI:** 10.1038/s41597-022-01286-6

**Published:** 2022-04-12

**Authors:** Guoxing Zhang, Yang Gao, Jiexun Li, Bin Su, Zhanglei Chen, Weichun Lin

**Affiliations:** 1 School of Management, Lanzhou University, Lanzhou, 730000 China; 2 Institute of Green Finance, Lanzhou University, Lanzhou, 730000 China; 3 Department of Decision Sciences, Western Washington University, Bellingham, WA 98225 USA; 4 Energy Studies Institute, National University of Singapore, Singapore, 119620 Singapore; 5 Department of Industrial Systems Engineering and Management, National University of Singapore, Singapore, Singapore

**Keywords:** Climate-change policy, Politics, Government

Correction to: *Scientific Data* 10.1038/s41597-022-01183-y, published online 11 March 2022

In this Data Descriptor, Yang Gao, Jiexun Li, Zhanglei Chen, and Weichun Lin were incorrectly denoted as corresponding authors. The correct corresponding authors are Guoxing Zhang and Bin Su. The original article has been corrected.

