# Supplementary material for: China’s environmental policy intensity for 1978–2019
Source: Sci Data. 2022 Mar 11;9:75. doi: 10.1038/s41597-022-01183-y (PMC8917127; doi:10.1038/s41597-022-01183-y)
Supplement: Supplementary file 1 — Supplementary Information [file 41597_2022_1183_MOESM1_ESM.docx]

**Supplementary Information**

R code:

shouji<-dir_or_file("E:/ ",special = "txt$")

fenciqi=worker(user = "E:/et.txt")

sink("E:/results.txt",append = FALSE,split = TRUE)

for (each in shouji) {

a=scancn(each)

b=gsub("(?i)[^\u4E00-\u9FA5]","",a)

c=slim_text(

b,

rm_place = TRUE,

rm_time = TRUE,

rm_eng = TRUE,

rm_alpha = TRUE

)

print(c)

}

sink()

fenci<-seg_file(

"E:/results.txt",

folder = "E:/ ",

mycutter=fenciqi

)

juzhen<-corp_or_dtm(

"E:/1 results.txt",

from = "dir",

type="dtm",

stop_word = "E: /stw.txt",

control = list(

wordLengths=c(2,100)

)

)

setwd("E:/191221-0428")

filelist<-Sys.glob("*.txt")

read.txt<-function(x){

des<-fread(x,header = FALSE,sep = " ",stringsAsFactors=F,encoding = "UTF-8",quote = "")

des<-t(des)

des<-as.data.frame(des)

}

review<-lapply(filelist,read.txt)

corpuslist<-function(des){

corpus<-VCorpus(VectorSource(des$V1))

}

corpus1<-lapply(review,corpuslist)

dtm<-function(corpus){

dtm1<-DocumentTermMatrix(corpus)

}

dtm2<-lapply(corpus1,dtm)

docs<-function(dtm1) {

docs1<-sapply(seq(1,nrow(dtm1)), toString)

}

docs2<-lapply(dtm2, docs)

matrix1<-function(dtm1) {

matrix2<-as.matrix(dtm1,rownames.value=seq(1,nrow(dtm1)))

}

matrix3<-lapply(dtm2, matrix1)

outpath<-"E:/191221-0428"

txtname=lapply(c("1"

),function(x) {

paste(x,".csv",sep="")

})

path<-sapply(txtname,function(x) {

paste(outpath,x,sep="/")

})

for (i in 1:length(matrix3)) {

write.csv(matrix3[[i]],file=path[i],row.names = F,fileEncoding = "UTF-8")

}

freq<-function(dtm1) {

freq1<-sort(colSums(as.matrix(dtm1)),decreasing = T)

wf1<-data.frame(word=names(freq1),freq=freq1)

}

freq2<-lapply(dtm2, freq)

outpath<-"E:/191221-0428"

wfname=lapply(c("1"

),function(x) {

paste(x,"wf.csv",sep="")

})

wfpath<-sapply(wfname,function(x) {

paste(outpath,x,sep="/")

})

for (i in 1:length(freq2)) {

write.csv(freq2[[i]],file=wfpath[i],row.names = F)

}

setwd("E:/191221-0428/wf")

filelist<-list.files(pattern = "*wf.csv",recursive = TRUE)

csv<-function(x){

a<-read.csv(x,header = T)

}

forall<-lapply(filelist,csv)

mydata<-list()

for (i in 1:length(forall)){

mydata[[i]]<-rename(forall[[i]],c(freq=filelist[[i]]))

}

setwd("E:/191221-0428/wf")

outpath<-"E:/191221-0428/wf"

nname=lapply("p",function(x){

paste(x,dir(),sep = "")

})

npath<-sapply(nname,function(x){

paste(outpath,x,sep="/")

})

for (i in 1:length(forall)){

write.csv(mydata[[i]],file=npath[i],row.names =T,col.names = T,quote = F)

}

setwd("E:/191221-0428/wf")

filelist1<-Sys.glob( "p*.csv")

d2<-read.csv("E:/191221-0428/wf/wf.csv",header = T)

for (i in 1:length(filelist1)){

a1<-read.csv(filelist1[i])

d2<-merge(a1,d2,by="word",all=TRUE,sort=TRUE)

}

write.csv(d2,"E:/191221-0428/wf/d2wf.csv",row.names = T,col.names = T,quote = F)

setwd("E:/191221-0428/wf")

filelist1y<-Sys.glob( "d2*.csv")

a2<-read.csv("E:/191221-0428/wf/final.csv",header=T)

a2<-as.data.frame(a2)

for (i in 1:length(filelist1y)){

a1<-read.csv(filelist1y[i],header = T)

a2<-merge(a1,a2,by="word",all=TRUE,sort=TRUE)

}

write.csv(a2,"E:/191221-0428/wf/final.csv",row.names = T,col.names = T,quote = F)

setwd("E:/191221-0428")

#final.csv = mtmatrix-r.csv

mt0501<-read.csv("E:/191221-0428/mtmatrix-r.csv",header = T)

mtscore<-read.csv("E:/191221-0428/mtscore.csv",header = T)

mtscore<-mtscore[,-c(1:3)]

mtscore<-as.data.frame(mtscore)

mtc<-cbind(mt0501,mtscore)

rownames(mtc)<-mtc[,1]

mtc<-mtc[,-1]

mtc[is.na(mtc)]<-0

write.csv(mtc,"E:/191221-0428/mtc.csv",row.names = T,col.names = T,quote = F)

attach(mtc)

mtx=mtc[,-25783]

mty=mtc[,25783]

zerovar1=nearZeroVar(mtx,freqCut = 99/1)

newdata6=mtx[,-zerovar1]

write.csv(newdata6,"E:/191221-0428/mt2-0502.csv",row.names = T,col.names = T,quote = F)

cora8<-cbind(newdata6,mty)

cora8<-round(cor(cora8),2)

cora8<-as.data.frame(cora8)

cora8=cora8[order(cora8[,1277],decreasing = T),]

write.csv(cora8,"E:/191221-0428/mtorder0502.csv",row.names = T,col.names = T,quote = F)

# form a specific lexicon = mtlist.csv

mtc<-fread("E:/191221-0428/mt/mtc.csv",header = T)

mtlist<-fread("E:/191221-0428/mt/mtlist.csv",header = T)

mtc1<-data.frame(t(mtc))

colnames(mtc1)<-mtc1[1,]

mtc2<-mtc1[-1,]

mtc2<-cbind(rownames(mtc2),mtc2)

mtc2<-dplyr::rename(mtc2,"word"="rownames(mtc2)")

mtmatrix<-merge(mtc2,mtlist,by="word",all = F)

write.csv(mtmatrix,"E:/191221-0428/mt/mtmatrix.csv",row.names = T,col.names = T,quote = F)

mtmatrix1<-read.csv("E:/191221-0428/mt/mtmatrix-score.csv",header = T)

rownames(mtmatrix1)<-mtmatrix1[,1]

mtmatrix1<-mtmatrix1[,-1]

mtmatrix1<-data.frame(t(mtmatrix1))

x2=mtmatrix1[,-476]

y2=mtmatrix1[,476]

inTrain=createDataPartition(y2,p=3/4,list=FALSE)

trainx2=x2[inTrain,]

testx2=x2[-inTrain,]

trainy2=y2[inTrain]

testy2=y2[-inTrain]

fitControl=trainControl(method = "repeatedcv",number = 10,repeats=3,returnResamp = "all")

metric<-"RMSE"

set.seed(1000)

fit.lm2<-train(trainx2,trainy2,method = "lm",metric = metric,trControl = fitControl)

set.seed(1000)

fit.ridge2=train(trainx2,trainy2,method="ridge",metric=metric,trControl=fitControl)

set.seed(1000)

fit.lasso2=train(trainx2,trainy2,method="lasso",metric=metric,trControl=fitControl)

set.seed(1000)

fit.rlm2=train(trainx2,trainy2,method="rlm",metric=metric,trControl=fitControl)

set.seed(1000)

fit.pls2=train(trainx2,trainy2,method="pls",metric=metric,trControl=fitControl)

set.seed(1000)

fit.glm2=train(trainx2,trainy2,method="glmnet",metric=metric,trControl=fitControl)

set.seed(1000)

fit.svm2<-train(trainx2,trainy2,method = "svmRadial",metric = metric,trControl = fitControl )

set.seed(1000)

fit.xgboost2<-train(trainx2,trainy2,method = "xgbLinear",metric = metric,trControl = fitControl)

fitControl=trainControl(method = "repeatedcv",number = 10,repeats=3,returnResamp = "all")

fit.rf2<-train(trainx2,trainy2,method = "rf",metric = metric,trControl = fitControl,localImp=TRUE)

result2<-resamples(list(lm2=fit.lm2,ridge2=fit.ridge2,lasso2=fit.lasso2,rlm2=fit.rlm2,pls2=fit.pls2,glm2=fit.glm2,svm2=fit.svm2,xgboost2=fit.xgboost2,rf2=fit.rf2))

sumresult<-summary(result2)

sumresult

prediction.lm2<-predict(fit.lm2,testx2)

mse.lm2=mean((prediction.lm2-testy2)^2)

print(mse.lm2)

rmse.lm2=sqrt(mse.lm2)

print(rmse.lm2)

mean(testy2)->mean.testy2

rsqu.lm2.ssr=sum((prediction.lm2-mean.testy2)^2)

rsqu.lm2.sse=sum((prediction.lm2-testy2)^2)

rsqu.lm2.sst=rsqu.lm2.sse+rsqu.lm2.ssr

rsqu.lm2=rsqu.lm2.ssr/rsqu.lm2.sst

print(rsqu.lm2)

prediction.ridge2<-predict(fit.ridge2,testx2)

mse.ridge2=mean((prediction.ridge2-testy2)^2)

print(mse.ridge2)

rmse.ridge2=sqrt(mse.ridge2)

print(rmse.ridge2)

rsqu.ridge2.ssr=sum((prediction.ridge2-mean.testy2)^2)

rsqu.ridge2.sse=sum((prediction.ridge2-testy2)^2)

rsqu.ridge2.sst=rsqu.ridge2.sse+rsqu.ridge2.ssr

rsqu.ridge2=rsqu.ridge2.ssr/rsqu.ridge2.sst

print(rsqu.ridge2)

prediction.lasso2<-predict(fit.lasso2,testx2)

mse.lasso2=mean((prediction.lasso2-testy2)^2)

print(mse.lasso2)

rmse.lasso2=sqrt(mse.lasso2)

print(rmse.lasso2)

rsqu.lasso2.ssr=sum((prediction.lasso2-mean.testy2)^2)

rsqu.lasso2.sse=sum((prediction.lasso2-testy2)^2)

rsqu.lasso2.sst=rsqu.lasso2.sse+rsqu.lasso2.ssr

rsqu.lasso2=rsqu.lasso2.ssr/rsqu.lasso2.sst

print(rsqu.lasso2)

prediction.rlm2<-predict(fit.rlm2,testx2)

mse.rlm2=mean((prediction.rlm2-testy2)^2)

print(mse.rlm2)

rmse.rlm2=sqrt(mse.rlm2)

print(rmse.rlm2)

rsqu.rlm2.ssr=sum((prediction.rlm2-mean.testy2)^2)

rsqu.rlm2.sse=sum((prediction.rlm2-testy2)^2)

rsqu.rlm2.sst=rsqu.rlm2.sse+rsqu.rlm2.ssr

rsqu.rlm2=rsqu.rlm2.ssr/rsqu.rlm2.sst

print(rsqu.rlm2)

prediction.pls2<-predict(fit.pls2,testx2)

mse.pls2=mean((prediction.pls2-testy2)^2)

print(mse.pls2)

rmse.pls2=sqrt(mse.pls2)

print(rmse.pls2)

rsqu.pls2.ssr=sum((prediction.pls2-mean.testy2)^2)

rsqu.pls2.sse=sum((prediction.pls2-testy2)^2)

rsqu.pls2.sst=rsqu.pls2.sse+rsqu.pls2.ssr

rsqu.pls2=rsqu.pls2.ssr/rsqu.pls2.sst

print(rsqu.pls2)

prediction.glm2<-predict(fit.glm2,testx2)

mse.glm2=mean((prediction.glm2-testy2)^2)

print(mse.glm2)

rmse.glm2=sqrt(mse.glm2)

print(rmse.glm2)

rsqu.glm2.ssr=sum((prediction.glm2-mean.testy2)^2)

rsqu.glm2.sse=sum((prediction.glm2-testy2)^2)

rsqu.glm2.sst=rsqu.glm2.sse+rsqu.glm2.ssr

rsqu.glm2=rsqu.glm2.ssr/rsqu.glm2.sst

print(rsqu.glm2)

prediction.svm2<-predict(fit.svm2,testx2)

mse.svm2=mean((prediction.svm2-testy2)^2)

print(mse.svm2)

rmse.svm2=sqrt(mse.svm2)

print(rmse.svm2)

rsqu.svm2.ssr=sum((prediction.svm2-mean.testy2)^2)

rsqu.svm2.sse=sum((prediction.svm2-testy2)^2)

rsqu.svm2.sst=rsqu.svm2.sse+rsqu.svm2.ssr

rsqu.svm2=rsqu.svm2.ssr/rsqu.svm2.sst

print(rsqu.svm2)

prediction.xgboost2<-predict(fit.xgboost2,testx2)

mse.xgboost2=mean((prediction.xgboost2-testy2)^2)

print(mse.xgboost2)

rmse.xgboost2=sqrt(mse.xgboost2)

print(rmse.xgboost2)

rsqu.xgboost2.ssr=sum((prediction.xgboost2-mean.testy2)^2)

rsqu.xgboost2.sse=sum((prediction.xgboost2-testy2)^2)

rsqu.xgboost2.sst=rsqu.xgboost2.sse+rsqu.xgboost2.ssr

rsqu.xgboost2=rsqu.xgboost2.ssr/rsqu.xgboost2.sst

print(rsqu.xgboost2)

prediction.rf2<-predict(fit.rf2,testx2)

mse.rf2=mean((prediction.rf2-testy2)^2)

print(mse.rf2)

rmse.rf2=sqrt(mse.rf2)

print(rmse.rf2)

rsqu.rf2.ssr=sum((prediction.rf2-mean.testy2)^2)

rsqu.rf2.sse=sum((prediction.rf2-testy2)^2)

rsqu.rf2.sst=rsqu.rf2.sse+rsqu.rf2.ssr

rsqu.rf2=rsqu.rf2.ssr/rsqu.rf2.sst

print(rsqu.rf2)

mse.result2<-list(lm=mse.lm2,ridge=mse.ridge2,lasso=mse.lasso2,rlm=mse.rlm2,pls=mse.pls2,glm=mse.glm2,svm=mse.svm2,xgboost=mse.xgboost2,rf=mse.rf2)

print(mse.result2)

rmse.result2<-list(lm=rmse.lm2,ridge=rmse.ridge2,lasso=rmse.lasso2,rlm=rmse.rlm2,pls=rmse.pls2,glm=rmse.glm2,svm=rmse.svm2,xgboost=rmse.xgboost2,rf=rmse.rf2)

print(rmse.result2)

rsqu.result2<-list(lm=rsqu.lm2,ridge=rsqu.ridge2,lasso=rsqu.lasso2,rlm=rsqu.rlm2,pls=rsqu.pls2,glm=rsqu.glm2,svm=rsqu.svm2,xgboost=rsqu.xgboost2,rf=rsqu.rf2)

print(rsqu.result2)

mtc1719<-fread("E:/191221-0428/mt/final1719.csv",header=T)

mtmatrix1719<-merge(mtc1719,mtlist,by=intersect(names(mtc1719)[1],names(mtlist)[1]))

write.csv(mtmatrix1719,"E:/191221-0428/mt/mtmatrix1719.csv",row.names = T,col.names = T,quote = F)

mtmatrix1719<-fread("E:/191221-0428/mt/mtmatrix1719.csv",header=T)

mtmatrix1719[is.na(mtmatrix1719)]<-0

mtmatrix1719<-data.frame(t(mtmatrix1719))

colnames(mtmatrix1719)<-mtmatrix1719[1,]

mtmatrix1719<-mtmatrix1719[-1,]

write.csv(mtmatrix1719,"E:/191221-0428/mt/mtmatrix17191.csv",row.names = T,col.names = T,quote = F)

mtmatrix17191<-apply(mtmatrix1719,2,as.numeric)

mtmatrix17191<-data.frame(mtmatrix17191)

mtmatrix17191<-cbind(mtmatrix17191,rownames(mtmatrix1719))

row.names(mtmatrix17191)<-mtmatrix17191[,476]

mtmatrix17191<-mtmatrix17191[,-476]

predict.rf2.1719<-predict(fit.rf2,mtmatrix17191)

predict.rf2.1719

write.csv(predict.rf2.1719,"E:/191221-0428/mt/predict1719.csv",row.names = T,col.names = T,quote = F)

prediction.rftrain2<-predict(fit.rf2,trainx2)

combine2<-cbind(prediction.rftrain2,trainy2)

combine2<-as.data.frame(combine2)

combine2<-plyr::rename(combine2,c("prediction.rftrain2"="predicted","trainy2"="observed"))

combine2s<-cbind(prediction.rf2,testy2)

combine2s<-as.data.frame(combine2s)

combine2s<-plyr::rename(combine2s,c("prediction.rf2"="predicted","testy2"="observed"))

predict.rf2.1719<-data.frame(predict.rf2.1719)

combine3<-plyr::rename(predict.rf2.1719,c("predict.rf2.1719"="predicted"))

mt.rf2<-rbind(combine2,combine2s)

write.csv(mt.rf2,"E:/191221-0428/mt/mtrf2.csv")

write.csv(combine3,"E:/191221-0428/mt/mtrf3.csv")
